# Delaying BCG immunotherapy onset after transurethral resection of non-muscle-invasive bladder cancer is associated with adverse survival outcomes

**DOI:** 10.1007/s00345-020-03522-3

**Published:** 2020-11-23

**Authors:** Wojciech Krajewski, Marco Moschini, Joanna Chorbińska, Łukasz Nowak, Sławomir Poletajew, Andrzej Tukiendorf, Luca Afferi, Jeremy Yuen-Chun Teoh, Tim Muilwijk, Steven Joniau, Alessandro Tafuri, Alessandro Antonelli, Francesco Cianflone, Andrea Mari, Ettore Di Trapani, Kees Hendricksen, Mario Alvarez-Maestro, Andrea Rodríguez-Serrano, Giuseppe Simone, Stefania Zamboni, Claudio Simeone, Maria Cristina Marconi, Riccardo Mastroianni, Guillaume Ploussard, Ekaterina Laukhtina, Karl Tully, Anna Kołodziej, Joanna Krajewska, Radosław Piszczek, Evanguelos Xylinas, Romuald Zdrojowy, European Association of Urology—Young Academic Urologists (EAU-YAU): Urothelial carcinoma working group

**Affiliations:** 1 Department of Urology and Oncologic Urology, Wrocław Medical University, Wroclaw, Poland; 2 Klinik Für Urologie, Luzerner Kantonsspital, Lucerne, Switzerland; 3 Second Department of Urology, Centre of Postgraduate Medical Education, Warsaw, Poland; 4 Department of Public Health, Wrocław Medical University, Wrocław, Poland; 5 S.H.Ho Urology, Department of Surgery, The Chinese University of Hong Kong, Hong Kong, China; 6 Department of Urology, University Hospitals Leuven, Leuven, Belgium; 7 Department of Urology, University of Verona, Azienda Ospedaliera Universitaria Integrata Verona, Verona, Italy; 8 Department of Urology, Careggi Hospital, University of Florence, Florence, Italy; 9 Department of Urology, IEO European Institute of Oncology, IRCCS, Milan, Italy; 10 Department of Urology, Netherlands Cancer Institute-Antoni Van Leeuwenhoek Hospital, Amsterdam, The Netherlands; 11 Department of Urology Hospital, Universitario La Paz Madrid, Madrid, Spain; 12 Department of Urology, Oncologic Urology “Regina Elena” National Cancer Institute, Via Elio Chianesi 53, 00144 Rome, Italy; 13 Urology Unit, ASST Spedali Civili, Brescia, Italy; 14 Department of Medical and Surgical Specialties, Radiological Science and Public Health, University of Brescia, Brescia, Italy; 15 Department of Urology, La Croix du Sud Hospital, Quint Fonsegrives, France; 16 Institute for Urology and Reproductive Health, Sechenov University, Moscow, Russia; 17 Department of Urology, Comprehensive Cancer Center, Medical University of Vienna, Vienna, Austria; 18 Department of Urology and Neurourology, Marien Hospital Herne, Ruhr-University Bochum, Herne, Germany; 19 Department and Clinic of Otolaryngology, Head and Neck Surgery, Wroclaw Medical University, Wroclaw, Poland; 20 Department of Urology and Oncologic Urology, Lowersilesian Specialistic Hospital, Wroclaw, Poland; 21 Department of Urology, Bichat-Claude Bernard Hospital, Assistance Publique-Hôpitaux de Paris, Paris Descartes University, Paris, France

**Keywords:** Bladder cancer, BCG, Time, Delay, Survival

## Abstract

**Purpose:**

This study was carried out to assess whether a prolonged time between primary transurethral resection of non-muscle-invasive bladder cancer (TURB) and implementation of bacillus Calmette–Guerin (BCG) immunotherapy (time to BCG; TTBCG) is associated with adverse oncological survival in patients with T1 high-grade (HG) non-muscle-invasive bladder cancer (NMIBC).

**Materials and methods:**

Data on 429 patients from 13 tertiary care centers with primary T1HG NMIBC treated with reTURB and maintenance BCG between 2001 and 2019 were retrospectively reviewed. Change-point regression was applied following Muggeo’s approach. The population was divided into subgroups according to TTBCG, whereas the recurrence-free survival (RFS) and progression-free survival (PFS) were estimated with log-rank tests. Additionally, Cox regression analyses were performed. Due to differences in baseline patient characteristics, propensity-score-matched analysis (PSM) and inverse-probability weighting (IPW) were implemented.

**Results:**

The median TTBCG was 95 days (interquartile range (IQR): 71–127). The change-point regression analysis revealed a gradually increasing risk of recurrence with growing TTBCG. The risk of tumor progression gradually increased until a TTBCG of approximately 18 weeks. When the study population was divided into two subgroups (time intervals: ≤ 101 and > 101 days), statistically significant differences were found for both RFS (*p* = 0.029) and PFS (*p* = 0.005). Furthermore, in patients with a viable tumor at reTURB, there were no differences in RFS and PFS. After both PSM and IPW, statistically significant differences were found for both RFS and PFS, with worse results for longer TTBCG.

**Conclusion:**

This study shows that delaying BCG immunotherapy after TURB of T1HG NMIBC is associated with an increased risk of tumor recurrence and progression.

**Electronic supplementary material:**

The online version of this article (10.1007/s00345-020-03522-3) contains supplementary material, which is available to authorized users.

## Introduction

Bacillus Calmette–Guérin (BCG) immunotherapy is a standard of care in high-risk non-muscle-invasive bladder cancer (NMIBC) [1, 2]. Despite the fact that BCG has been used for decades, many therapeutic details still remain unclear. One of these details is the upper time limit to which BCG may be safety delayed after transurethral resection.

To reduce the risk of BCG complications, the European Association of Urology (EAU) guidelines recommend the onset of BCG immunotherapy at least 2 weeks after transurethral resection [1]. However, no upper time limit is specified. Fundamental BCG studies were carried out to follow the accrual protocol of BCG implementation 7–14 days after primary transurethral resection of non-muscle-invasive bladder cancer (TURB) [3, 4]. Yet, in real life, due to the pathological assessment time, necessity of reTURB performance, patient insurance status, waiting lists, BCG shortage, and various other logistic reasons, it is almost impossible to begin BCG immunotherapy in a fortnight.

In this study, the main hypothesis was that a prolonged time between operative treatment and BCG implementation (time to BCG; TTBCG) would be associated with adverse oncological survival.

## Material and methods

The current study was approved by an institutional review board for institutional data sharing from all participating sites. We retrospectively reviewed data on 429 patients from 13 tertiary care centers with primary T1HG NMIBCs with or without concomitant carcinoma in situ (CIS) treated with reTURB before the BCG induction course and further maintenance BCG immunotherapy between 2001 and 2019.

All patients were treated with BCG immunotherapy induction and maintenance courses. The BCG instillations were administered according to the international guidelines and local protocols at the time. Each patient included in the analysis received a minimum of five instillations of the induction course and two instillations of the maintenance course [5].

Specimens were evaluated by dedicated uropathologists in each participating center, and no central assessment was applied. Patients were followed up according to EAU guidelines at the time.

Concomitant CIS was defined as the coexistence of carcinoma in situ in conjunction with the exophytic tumor. Recurrence was defined as recurrence of a tumor of any stage and grade confirmed by TURB and histologic assessment. A viable tumor at reTURB and tumor recurrence in the upper urinary tract were not considered as recurrence. Progression was defined as tumor relapse at tumor stage T2 or higher in the bladder, stromal invasion of the prostatic urethra, or distant (e.g., in the lymph nodes) progression. Patients with T2 lesions at reTURB were not included in the analysis.

The primary database was constructed using cases from 13 centers and included 1511 high-risk NMIBC patients. The study analysis exclusion criteria were as follows: incomplete data on major variables, tumors other than T1 high-grade (HG) tumors, recurrent tumors, incomplete primary TURB with evident residual disease, reTURB performed after BCG implementation, time interval between TURB and reTURB > 90 days, time interval between reTURB and BCG onset > 90 days, number of BCG instillations < 7, follow-up < 6 months, and any dose other than a full one of BCG for a given strain. After the exclusion process, 429 cases underwent further analysis.

### Statistical analyses

Change-point regression was applied following Muggeo’s approach [6]. The study population was divided into subgroups on the basis of time intervals between primary TURB and BCG onset, which were then compared using chi-square and Mann–Whitney tests. The recurrence-free survival (RFS) and progression-free survival (PFS) were estimated using the log-rank method, and Kaplan–Meier curves were plotted. Additionally, Cox regression analyses were performed for both RFS and PFS. Due to differences in baseline patient characteristics in both groups, we used a 1:1 propensity-score-matched analysis (PSM) adjusted for gender, smoking status, age, presence of MP in the primary specimen, tumor focality and size, incidence of concomitant CIS, and reTURB status [7]. Additionally, to reduce the bias of unweighted estimators and adjust for covariate imbalance between treatment groups without losing patients, we performed inverse-probability weighting (IPW) using the same variables as in PSM [8].

Patients without an event or who died before an event were censored on the last date of follow-up. Times to events were calculated by taking the date of primary resection as time zero. Statistical significance was considered at *p* < 0.05. Statistical analyses were performed using STATA 14 (Stata Corp., College Station, TX, USA) and the R platform (R project, Vienna, Austria).

## Results

Baseline patient characteristics are presented in Table 1. The median time from primary resection to reTURB was 40 days (interquartile range (IQR): 28–52), the median time from primary resection to BCG administration was 95 days (IQR: 71–127), and the median difference between reTURB and BCG onset was 56 days (IQR: 35–79).

**Table 1:** The patient baseline characteristics (*χ*^2^ and Mann–Whitney test *p* values of the differences between the two study groups)

	All patients (*n* = 429)	TTBCG ≤ 101 days (*n* = 200; 46,6%)	TTBCG > 101 days (*n* = 229; 53,4%)	*p* value
Age (median; IQR)	67,1; 58–75	67,1; 58–74	67,0; 58–75	0.561
Gender (M/F)	349/80 (81.4/18.6%)	161/39 (80.5/19.5%)	188/41 (82,1/17,9%)	0.672
Smoking history; *n* (%)				0.284
Never	138 (32.2%)	68 (34%)	70 (30.6%)	
Former	179 (41.7%)	75 (37.5%)	104 (45.4%)	
Current	103 (24%)	51 (25.5%)	52 (22.7%)	
UKN	9 (2.1%)	6 (3%)	3 (1.3%)	
Concomitant CIS				0.479
Yes	75 (17.5%)	33 (16.6%)	42 (18.3%)	
No	352 (82.1%)	167 (83.5%)	185 (80.8%)	
UKN	2 (0.5%)		2 (0.9%)	
Tumor size				0.152
< 3 cm	197 (45.9%)	99 (49.5)	98 (42.8%)	
≥ 3 cm	201 (46,9%)	84 (42%)	117 (51,1%)	
UKN	31 (7.2%)	17 (8.5%)	14 (6.1%)	
Tumor focality				0.472
Solitary	191 (44.5%)	93 (46.5%)	98 (42.8%)	
Multiple	214 (49.9%)	94 (47%)	120 (52.4%)	
UKN	24 (5.6%)	13 (6.5%)	11 (4.8%)	
Muscularis propria in the primary specimen (yes/no)	307/100/22 (71.6/23.3/5.1%)	144/43/13 (72/21.5/6.5%)	163/57/9 (71.2/24.9/3.9%)	0.385
Residual disease at reTURB (yes/no)	163/266 (38/62%)	62/138 (31/69%)	101/128 (44.1/55.9%)	**0.005**
Muscularis propria in the reTURB specimen				0.925
Yes	286 (66.7%)	134 (67%)	152 (66.4%)	
No	123 (28.7%)	56 (28%)	67 (29.3%)	
UKN	20 (4.7%)	10 (5%)	10 (4.4%)	
BCG strain; n (%)				**0.019**
Moreau	99 (23.1%)	58 (29%)	41 (17.9%)	
TICE	169 (39.4%)	66 (33%)	103 (45%)	
RIVM	118 (27.5%)	54 (27%)	64 (27.9%)	
Other	43 (10%)	22 (11%)	21 (9.2%)	
Total number of BCG instillations (median; IQR)	15; 9–18	15; 9–18	15; 9–16	0.211
Observation time – months (median; IQR)	40; 24–58	36,1; 23–56	43,9; 25–62	**0.023**
Recurrence	144 (33.6%)	59 (29.5%)	85 (37.1%)	0.096
Progression	61 (14.2%)	37 (18.5%)	24 (10.5%)	**0.018**
Cancer specific death	33 (7.7%)	19 (9.5%)	14 (6.1%)	0.189

The value of adjusted *p* < 0.05 was considered statistically significant (bolded)

*IQR* interquartile range, *M* male, *F* female, *CIS* carcinoma in situ, *UKN* unknown

The change-point regression analysis revealed a gradually increasing risk of recurrence with growing TTBCG; however, no significant marginal time point was found after which the risk of recurrence increased or decreased statistically. In the case of tumor progression analysis, the risk gradually increased until a TTBCG of approximately 18 weeks (129 days). Moreover, 18 weeks after primary TURB, further postponement of BCG was not associated with an increased risk of progression.

The study population was divided into four groups on the basis of the TTBCG (6–10, 11–14, 15–18, and 19–25 weeks), comparable in terms of the number of patients. Furthermore, classical Cox regression analysis was performed. The results in Table 2 show a significant hazard increase in the number of analyzed clinical events in patients with a longer TTBCG.

**Table 2:** Detailed analysis of influence of reTURB timing on oncological outcomes

Clinical event	Group (TTBCG intervals, weeks)	HR	CI95%	*p*value
Recurrence	6–10	1.00	Ref.	
	11–14	1.14	(0.70.1.86)	0.590
	15–18	1.54	(0.94.2.50)	0.084
	19–25	1.62	(1.02.2.58)	**0.041**
Progression	6–10	1.00	Ref.	
	11–14	1.94	(0.78.4.81)	0.152
	15–18	3.15	(1.32.7.55)	**0.010**
	19–25	3.34	(1.42.7.81)	**0.005**

The value of adjusted *p* < 0.05 was considered statistically significant (bolded)

*HR* Hazard ratio, *TTBCG* time to BCG, *95%CI* 95% confidence interval

**Fig. 1:**
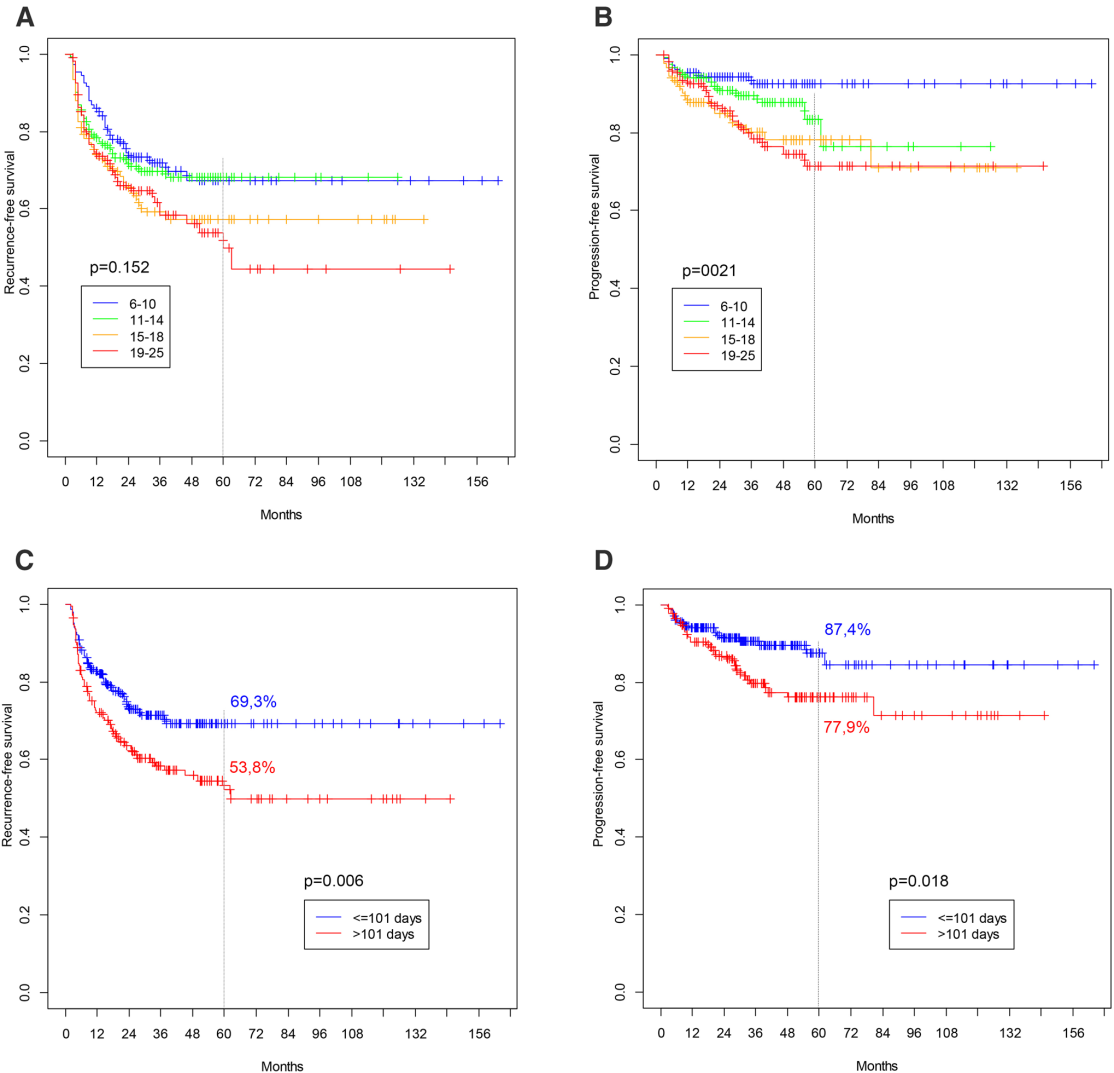
Survival curves for analysed subgroups. **a** Recurrence-free survival (TTBCG intervals: 6–10; 11–14; 15–18 and 19–25 weeks) (*p* = 0.152). **b** Progression-free survival (TTBCG intervals: 6–10; 11–14; 15–18 and 19–25 weeks) (*p* = 0.021). **c** Recurrence-free survival (TTBCG intervals: ≤ 101 and > 101 days) (*p* = 0.006). **d** Progression-free survival (TTBCG intervals: 101 and > 101 days) (*p* = 0.018)

Figure 1a, b demonstrate survival curves for both RFS and PFS for the four analyzed subgroups (TTBCG intervals: 6–10, 11–14, 15–18, and 19–25 weeks). The differences in survival were statistically significant for PFS (*p* = 0.021), with worse results for groups with a longer time interval. For RFS, no significant differences were noted (*p* = 0.152). Because of the relatively low and uneven number of events in each group, the whole population was subsequently divided into two main subgroups, with a marginal value of 101 days (mean TTBCG value for whole study population). Figure 1c, d present the survival curves for both RFS and PFS for these two analyzed subgroups (time intervals: ≤ 101 and > 101 days). Statistically significant differences were found for both RFS (*p* = 0.029) and PFS (*p* = 0.005).

In the subgroup of patients showing no viable tumor at reTURB, similar results could be observed, with a TTBCG ≤ 101 days being associated with favorable RFS and PFS (*p* = 0.007 and *p* = 0.005, respectively; Online Resource 1a, b). In contrast, in patients with a viable tumor at reTURB, there were no differences in RFS and PFS (Online Resource 1c, d).

**Fig. 2:**
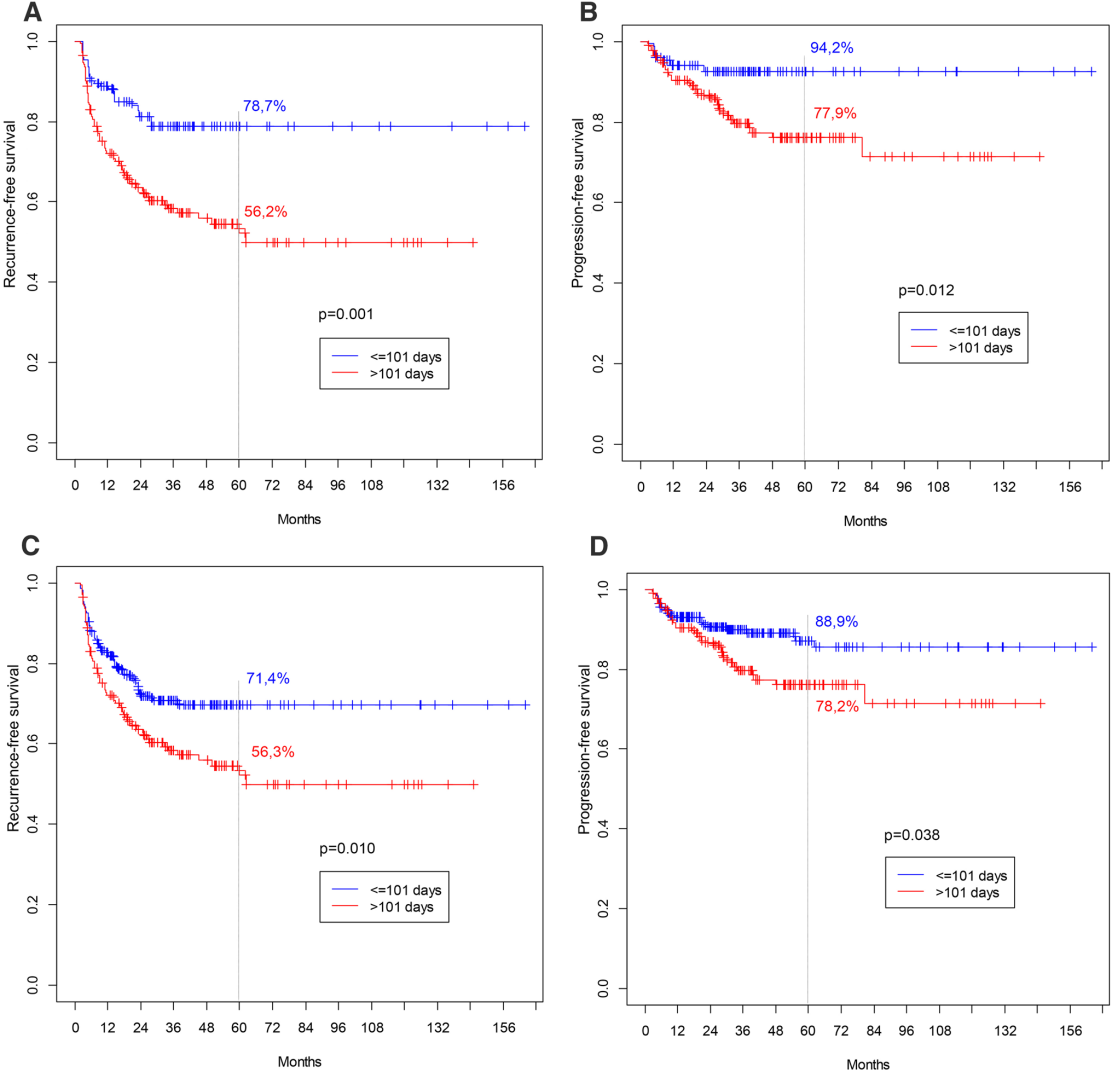
**a** Recurrence-free survival for 5 years follow-up after PSM (*p* = 0.001). **b** Progression-free survival for 5 years follow-up after PSM (*p* = 0.012). **c** Recurrence-free survival for 5 years follow-up after IPW (*p* = 0.010). **d** Progression-free survival for 5 years follow-up after IPW (*p* = 0.038)

Because of the retrospective and multicenter nature of the study, some disparities in baseline patient characteristics were observed between subgroups; therefore, PSM and IPW were performed. After matching, 270 patients were included in the analysis (135 in each group). After PSM, statistically significant differences were found for both RFS and PFS, with worse results seen for patients with a longer TTBCG (Fig. 2a, b). Similarly, when IPW was employed, clearly significant differences were observed for both RFS and PFS (Fig. 2c, d).

## Discussion

To the best of our knowledge, the influence of the time period between surgical treatment and BCG induction on oncological results was not previously analyzed in a setting involving real patients. Therefore, the clinical practice of introducing BCG “soon” after transurethral resections is instead based on clinical experience, common sense, and the extrapolation of results from studies analyzing similar issues in other neoplasms [9, 10]. In early 2013, Rentsch et al. published a study investigating several clinical parameters and their impact on the optimal protocol of BCG immunotherapy in a theoretical, mathematical setting [11]. The authors showed that a shorter interval between surgery and BCG therapy is associated with a greater chance of achieving a clinical response. It could be hypothesized that the immunogenic effect of BCG differs with the time period following surgical resection; alternatively, the tumor may have increased time for development with a longer time period.

In this study, a retrospective group of patients with primary T1HG NMIBCs was analyzed. It was demonstrated that the risk of recurrence and progression was dependent on TTBCG. The progression risk gradually increased until a TTBCG of approximately 18 weeks. In the recurrence analysis, the increase in risk was analogous to that seen in the progression analysis; however, no specific change point was found.

The population was divided into four subgroups on the basis of TTBCG intervals (6–10, 11–14, 15–18, and 19–25 weeks) to precisely determine the effect of TTBCG on survival. It was shown that, when compared to the group that received BCG earliest, postponing BCG onset to more than 15 weeks was associated with an increased risk of both clinical events (by at least 50 and 200% for every week of delay for recurrence and progression, respectively). However, as categorization into four groups resulted in a small number of clinical events in each, we finally split the whole population into two comparable subgroups on the basis of the mean value of TTBCG (TTBCG intervals: ≤ 101 and > 101 days). The latter population was followed up over a statistically longer time period and was found to have more tumors at reTURB. The residuals did not differ statistically in terms of tumor stage between groups (data not shown). This may be explained by the fact that the time period between primary TURB and reTURB was on the verge statistical significance (*p* = 0.098; data not shown) between the groups with TTBCG intervals of ≤ 101 and > 101 days. When a simple comparison was performed, clear differences in both RFS and PFS were observed, with better survival for the group with a TTBCG of ≤ 101 days (Fig. 1). It is worth mentioning that when a separate analysis was performed for patients with a viable tumor at reTURB, the effect of TTBCG was no longer visible. Furthermore, both RFS and PFS were rather unfavorable when compared to patients with a negative reTURB in the group with a TTBCG of ≤ 101 days (Online Resource 1). These findings contribute new information to the previously known fact that patients with residual tumors at reTURB are characterized with lower survival when compared to those with a negative reTURB; thus, the follow-up schedule in these patients should be more thorough [12, 13].

Finally, to avoid potential bias resulting from subgroup disparities, Kaplan–Meier analysis was performed after PSM and IPW adjusting for basic patient characteristics. Once more, significant differences were noted for both RFS and PFS, with better survival found for the group with a TTBCG of ≤ 101 days.

### Study limitations

Notwithstanding its several strengths, our study suffered from some limitations. First, most data were collected retrospectively. However, when compared with recently published high-quality data, this study population is fairly representative in terms of basic characteristics [14]. Recurrence and progression rates may seem low when compared with the classic EORTC or better CUETO nomograms; however, once more, our results did not significantly differ from contemporary series [15]. Conclusively, to overcome the limitations of the study’s retrospective design, we performed PSM and IPW analysis, matching patients for baseline characteristics. Second, to preserve the homogeneity of the population, we included only patients that received at least five induction and two maintenance instillations, representing adequate BCG exposure. However, as a result, some relevant patients (e.g., patients with a poor outcome at reTURB after BCG induction) were not included. Third, the adoption of long time periods between oncological treatment components as an inclusion criterion may have resulted in some clinical events happening unnoticed and being faultily classified. However, according to the accessible literature and available data, a significant postponement of therapeutic procedures takes place in “real-life” clinical practice. Fourth, we conducted no central specimen review and no T1 tumor substage analysis. Fifth, all procedures were performed at high-volume oncological centers. As a result, our findings may not be applicable to centers having less experience with bladder cancer treatment. Lastly, data regarding the experience of surgeons, technical details (e.g., en bloc, reTURB range), World Health Organization (WHO) 1973 grade, immediate single-instillation chemotherapy, LVI, VH, and prostatic involvement of the tumors were not uniformly reported and/or unreliable; therefore, they were not included in the analysis.

## Conclusions

This study showed that delaying BCG immunotherapy after TURB of T1HG NMIBC is associated with an increased risk of tumor recurrence and progression. However, in patients with a positive reTURB, we found that the timing of BCG did not impact tumor recurrence and progression.

## Electronic supplementary material

Below is the link to the electronic supplementary material.

Supplementary file1 (PDF 255 KB)
