# Supplementary material for: Delaying BCG immunotherapy onset after transurethral resection of non-muscle-invasive bladder cancer is associated with adverse survival outcomes
Source: World J Urol. 2020 Nov 23;39(7):2545–52. doi: 10.1007/s00345-020-03522-3 (PMC8332577; doi:10.1007/s00345-020-03522-3)
Supplement: Supplementary file 1 — Supplementary file1 (PDF 255 KB) [file 345_2020_3522_MOESM1_ESM.pdf]

Delaying BCG immunotherapy onset after transurethral resection of non-muscle-invasive bladder cancer is associated with adverse survival outcomes.

World Journal of Urology

Wojciech Krajewski1, Marco Moschini2, Joanna Chorbińska1, Łukasz Nowak1, Sławomir Poletajew3, Andrzej Tukiendorf4, Luca Afferi2, Jeremy Yuen-Chun Teoh5, Tim Muilwijk6, Steven Joniau6, Alessandro Tafuri7, Alessandro Antonelli7, Francesco Cianflone7, Andrea Mari8, Ettore Di Trapani9, Kees Hendricksen10, Mario Alvarez-Maestro11, Andrea Rodríguez Serrano11, Giuseppe Simone12, Stefania Zamboni13, Claudio Simeone13, Maria Cristina Marconi13, Riccardo Mastroianni12, Guillaume Ploussard14, Ekaterina Laukhtina15,16, Karl Tully17, Anna Kołodziej1, Joanna Krajewska18, Radosław Piszczek19, Evanguelos Xylinas20, Romuald Zdrojowy1,

1. Department of Urology and Oncologic Urology, Wrocław Medical University, Wroclaw, Poland
2. Klinik für Urologie, Luzerner Kantonsspital, Lucerne, Switzerland
3. Second Department of Urology, Centre of Postgraduate Medical Education, Warsaw, Poland
4. Department of Public Health, Wrocław Medical University, Wrocław, Poland
5. S.H.Ho Urology, Department of Surgery, The Chinese University of Hong Kong, Hong Kong, China
6. Department of Urology, University Hospitals Leuven, Leuven, Belgium.
7. Department of Urology, University of Verona, Azienda Ospedaliera Universitaria Integrata Verona, Verona, Italy
8. Department of Urology, Careggi Hospital, University of Florence, Florence, Italy
9. Department of Urology, IEO European Institute of Oncology, IRCCS, Milan, Italy
10. Department of Urology, Netherlands Cancer Institute-Antoni Van Leeuwenhoek Hospital, Amsterdam, The Netherlands
11. Department of Urology Hospital Universitario La Paz Madrid Spain
12. Oncologic Urology "Regina Elena" National Cancer Institute Department of Urology Via Elio Chianesi 53, 00144, Rome, Italy
13. Urology Unit, ASST Spedali Civili, Brescia, Italy; Department of Medical and Surgical Specialties, Radiological Science and Public Health, University of Brescia, Italy

14. Department of Urology, La Croix du Sud Hospital, Quint Fonsegrives, France
15. Institute for Urology and Reproductive Health, Sechenov University, Moscow, Russia
16. Department of Urology, Comprehensive Cancer Center, Medical University of Vienna, Vienna, Austria
17. Department of Urology and Neurourology, Marien Hospital Herne, Ruhr-University Bochum, Herne, Germany
18. Department and Clinic of Otolaryngology, Head and Neck Surgery, Wroclaw Medical University
19. Department of Urology and Oncologic Urology, Lowersilesian Specialistic Hospital, Wroclaw, Poland
20. Department of Urology, Bichat-Claude Bernard Hospital, Assistance Publique-Hôpitaux de Paris, Paris Descartes University, Paris, France.

On behalf of European Association of Urology- Young Academic Urologists (EAU-YAU): Urothelial carcinoma working group

Corresponding author: Wojciech Krajewski, MD PhD; Department of Urology and Oncological Urology, Wrocław Medical University, Wrocław, Poland Tel/fax: 0048717331010, Email: wk@softstar.pl, ORCID: https://orcid.org/0000-0003-1727-2283

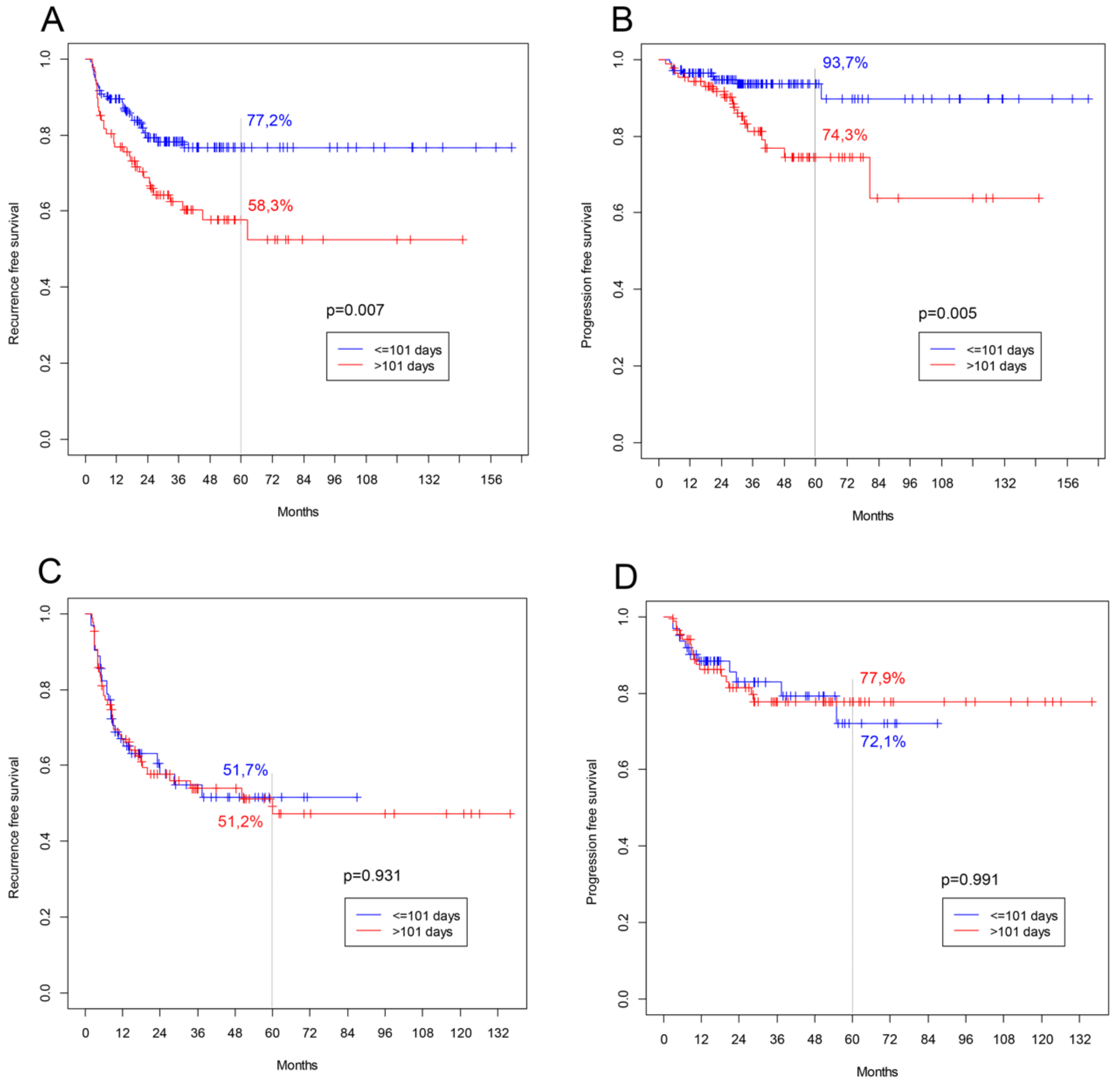

Online Resource 1 a. Recurrence-free survival for patients with negative reTURB (p=0.007). b. Progression-free survival for patients with negative reTURB (p=0.005). c. Recurrence-free survival for patients with positive reTURB (p=0.931). d Progression-free survival for patients with positive reTURB (p=0.991)
